# Computational Strategies and Algorithms for Inferring Cellular Composition of Spatial Transcriptomics Data

**DOI:** 10.1093/gpbjnl/qzae057

**Published:** 2024-08-07

**Authors:** Xiuying Liu, Xianwen Ren

**Affiliations:** Changping Laboratory, Beijing 102206, China; Changping Laboratory, Beijing 102206, China

**Keywords:** Spatial transcriptomics, Single-cell sequencing, Cellular composition, Spot deconvolution, Cell type decomposition

## Abstract

Spatial transcriptomics technology has been an essential and powerful method for delineating tissue architecture at the molecular level. However, due to the limitations of the current spatial techniques, the cellular information cannot be directly measured but instead spatial spots typically varying from a diameter of 0.2 to 100 µm are characterized. Therefore, it is vital to apply computational strategies for inferring the cellular composition within each spatial spot. The main objective of this review is to summarize the most recent progresses in estimating the exact cellular proportions for each spatial spot, and to prospect the future directions of this field.

## Introduction

**Figure 1:**
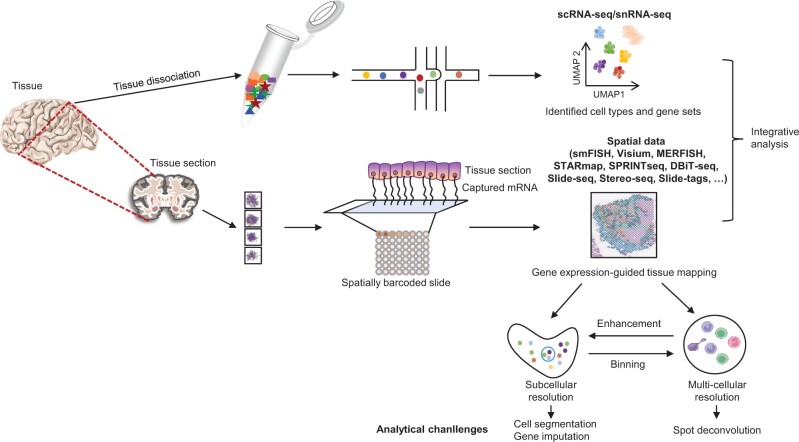
Schematic chart of the experimental and analytical workflow of typical studies applying scRNA-seq and spatial transcriptomics techniques scRNA-seq, single-cell RNA sequencing; snRNA-seq, single-nucleus RNA sequencing; smFISH, single-molecule fluorescence *in situ* hybridization; MERFISH, multiplexed error-robust fluorescence *in situ* hybridization; STARmap, spatially resolved transcript amplicon readout mapping; SPRINTseq, Spatially Resolved and signal-diluted Next-generation Targeted sequencing; DBiT-seq, deterministic barcoding in tissue for spatial omics sequencing; mRNA, messenger RNA; UMAP, Uniform Manifold Approximation and Projection.

Single-cell RNA sequencing (scRNA-seq) technologies have been a powerful tool to capture gene expression at a cellular resolution during the past decade [1,2]. However, to reach single-cell resolution, tissues need to be dissociated in most of scRNA-seq methods, and thus the spatial organization of cells within a tissue is lost [3]. However, spatial structure of the cells is critical to study the biological functions of genes and individual cells from a holistic view to reveal the spatial pattern of gene expression, the individual cellular states and their cellular microenvironment, and the heterogeneous cell–cell interactions [4–6]. While spatially resolved transcriptomics technologies, such as spatial transcriptomics (ST), Slide-seq, 10X Genomics Visium, high-definition spatial transcriptomics (HDST), deterministic barcoding in tissue for spatial omics sequencing (DBiT-seq), Spatially Resolved and signal-diluted Next-generation Targeted sequencing (SPRINTseq), Stereo-seq, and Slide-tags, have been developed to capture gene expression spatially across the tissue sections, they are typically limited to profiling small regions or spots with mixtures of multiple cells from the same or different cell types [7–14] (**Figure 1**). Therefore, uncovering the cellular composition of the spatial spots with mixed cells at each tissue location is currently a critical task to understand the molecular and cellular architecture of tissues at resolution close to single cells [15]. Various computational strategies and algorithms have been proposed and benchmarked to infer the composition of cell types at each spatial spot [16–25]. The aim of this work is to review the recent progress in this field from an algorithmic perspective and with the expectation to provide guidelines for the choice of methods under different scenarios and shed light into future developing directions.

## The conceptual framework of cellular composition inference of ST data

As a computational biology question, the conceptual framework of cellular composition inference includes three basis components: (1) the ST data as the input; (2) cellular definitions; and (3) the spot-by-cell matrix as the output. These three components form the basic elements of all strategies and algorithms for cellular composition inference and define the characteristics of different strategies and methods. Because multiple ST technologies are available, each of which has distinct technological features, the current ST data can be grouped into two types according to spatial resolution. For ST data with spatial resolution larger than 10 μm, each ST spot may be comprised of multiple cells. These ST spots form the major data type that we discuss in this review. For ST data with spatial resolution smaller than 1 μm, one cell may be comprised of dozens of or even hundreds of spots, and the cellular composition inference question for such type of ST data is generally defined on larger regions that include dozens of or hundreds of spatially adjacent spots. Otherwise, the computational biology question that infers the cellular identity of a spatially continuous region of spots is generally referred to cell segmentation (Figure 1). Typical algorithms include Cellpose [26] and Baysor [27]. In this review, we do not discuss algorithms for cell segmentation because of the inherently distinct logical structure.

**Figure 2:**
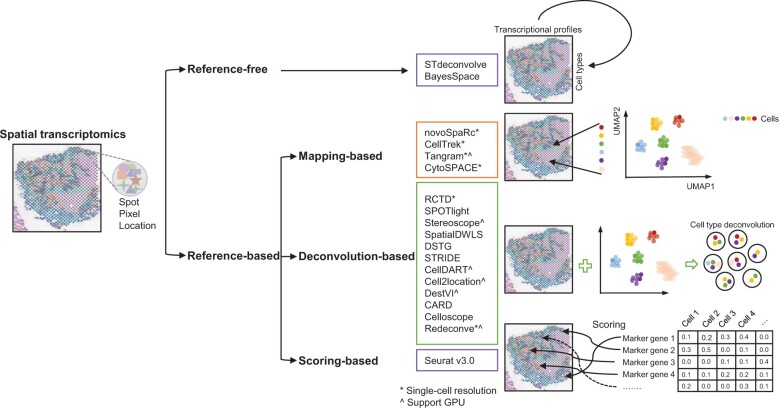
The current algorithms and methods for spatial transcriptomics data analysis GPU, graphics processing unit.

According to the characteristics of the second and third components, we can classify the current algorithms into three major groups: (1) the scoring strategy, which defines a scoring function based on signature genes of different cell types and the gene expression profiles of individual spots and outputs a spot-by-cell type matrix with scores as elements; (2) the mapping strategy, which defines a distance/similarity function between the gene expression profiles of single cells and the gene expression profiles of individual spots and outputs a spot-by-cell matrix that is better constrained on single cells; and (3) the deconvolution strategy, which defines a regression function between the gene expression profiles of individual spots and a set of single cells and outputs a spot-by-cell type matrix that is better constrained on spots. Each strategy has multiple algorithms and tools developed (**Figure 2**).

In general, functions implemented by the scoring strategy are weakly defined, and thus the outputs generated by the scoring strategy requires more domain knowledge and professional interpretation. Therefore, we do not include algorithms applying the scoring strategy to analyze ST data in this review. Here, we only introduce Seurat [28], one of the most popular tools for single-cell and ST analyses, developed by the Satija Lab. Seurat is designed for quality control, analysis, and exploration of scRNA-seq data, but it has been extended to handle ST data. Seurat v3.0 and later versions integrate various “scoring strategies” that can be used to summarize and interpret spatial gene expression patterns. In contrast, the mapping and deconvolution strategies impose more constraints on the algorithms, and the biological meanings of the outputs are more definite [29]. Mapping-based constraints are generally imposed via distance/similarity functions, which have the power to conduct the mapping operation at single-cell resolution. However, the mapping results may be incorrect when the spot-to-cell relationship goes complex. Different from the mapping strategy, the deconvolution strategy views the question from a regression perspective, which imposes constraints on individual spots and a set of single cells. The regression perspective enables reconstruction of the gene expression profiles of spots based on different composition of single cells, and thus is more faithful to ST observations. The difficulty of deconvolution analysis lies in the resolution because co-linearity will occur among the gene expression profiles of closely similar single cells, which will result in computational uncertainty. Taking into consideration this specific perspective, we have reviewed the existing algorithms for inferring cellular composition from ST data, while acknowledging that our literature search may not be exhaustive. We apologize for any omission of significant studies resulting from this limited scope.

## Mapping-based algorithms for inferring cellular composition of ST data

In recent years, different mapping-based approaches of spatial spot deconvolution have been developed based on statistical, machine learning, and deep learning frameworks [24]. Most tools require the count table of gene expression from scRNA-seq and spatial spots as input, sometimes also require the spatial coordinates and ST spots, and give us the matrix of cell type proportion or compositions. These tools include novoSpaRc [30], CellTrek [31], Tangram [32], and cellular (Cyto) Spatial Positioning Analysis via Constrained Expression alignment (CytoSPACE) [33]. Here, we summarize the characteristics of these tools in Table S1 and briefly comment their design, implementation, features, and performance below (Figure 2; Table S1).

### novoSpaRc

novoSpaRc is a computational framework that probabilistically assigns single cells to a tissue’s physical structure, which can be a geometry specified by users or a ST map [30,34]. When a geometry is provided, novoSpaRc can infer gene expression patterns across the geometry. Therefore, novoSpaRc is also assumed to be an algorithm that *de novo* reconstructs cellular spatial organization based on scRNA-seq data, similar to CSOmap, another spatial *de novo* reconstructing algorithm [35]. Different from CSOmap, which assumes that cellular spatial organization is formed via cellular self-assembly mediated by ligand–receptor interactions, novoSpaRc assumes that cells of similar gene expression profiles have close spatial relationship. Therefore, novoSpaRc and CSOmap fit different applications. As a ST data analyzing algorithm, novoSpaRc considers a gene expression matrix and the coordinates of the physical space as inputs and outputs a transport matrix encoding a probabilistic mapping of cells onto the target space locations. Because of the assumption that cells sharing similar expression profiles are also physically close to each other, the outputs of ST data analysis will generally show a continuous pattern. Algorithmically, novoSpaRc establishes the computational framework by optimal transport, a classical computational question in the operation research field. novoSpaRc uses biological assumptions to place cells with similar gene expression profiles close together and is flexible in reconstruction, yet it may struggle with accuracy for non-conforming tissues and is computationally intensive for large datasets.

### CellTrek

CellTrek is a computational toolkit that establishes a function between gene expression profiles and physical coordinates, which enables projecting single cells to the physical space in tissue sections based on scRNA-seq data [31]. First, CellTrek integrates scRNA-seq and ST data into a shared domain to reduce technological biases. Then, CellTrek predicts the spatial coordinates by a multivariate random forest (RF) model trained by using the shared features between ST and scRNA-seq data after removing technological biases. The trained model is applied to the shared features, and derives an RF distance matrix that measures the expression similarity between ST spots and single cells. Based on the RF distance matrix, CellTrek generates a sparse spot–cell graph using mutual nearest neighbors (MNNs) and obtains spatial coordinates for individual cells from their neighbor spots. In principle, the coordinates of single cells may locate within the circles of spots or the space among spots and the number of cells falling into the same spot may vary from zero to a large digit. CellTrek integrates scRNA-seq and ST data using RF to reduce biases and provides realistic spatial contexts, but it can be computationally demanding and less scalable.

### Tangram

Tangram employs a deep learning framework to map single-cell profiles generated by scRNA-seq or single-nucleus RNA sequencing (snRNA-seq) data to spatial spots with multiple objectives [32]. In addition to the objective that maximizes the similarity of single cells with spatial spots, Tangram also requires optimizing the similarity of spatial distribution between observed and reconstructed gene expression profiles. While such additional objectives will cost more computational resources, the alignment accuracy between scRNA-seq and ST data should be further improved. Besides, Tangram allows users to make use of various kinds of prior knowledge such as cell number in each spatial location to infer the cellular composition in each spatial spot. Tangram can tackle different ST scenarios, expanding limited ST signatures to genome-wide patterns for high-resolution targeted data [*e.g.*, single molecule fluorescence *in situ* hybridization (smFISH), multiplexed error-robust fluorescence *in situ* hybridization (MERFISH), and spatially resolved transcript amplicon readout mapping (STARmap)], generating single-cell resolution for lower-resolution spatial platform (*e.g.*, 10X Genomics Visium), correcting the low-accuracy expression for high-throughput but lower-accuracy datasets, and yielding spatial patterns and multimodal maps for multimodal single-cell dataset. Tangram employs a deep learning framework to optimize cell–spot similarity, allowing for the integration of prior knowledge, but requires significant computational resources and can be complex to use.

### CytoSPACE

CytoSPACE is an efficient computational method for aligning individual cells from a scRNA-seq dataset to precise spatial spots via convex linear optimization [33]. CytoSPACE considers single-cell assignment as a convex optimization problem and solves it by the Jonker–Volgenant shortest augmenting path algorithm. This method guarantees an optimal alignment result while exhibiting improved noise tolerance. First CytoSPACE estimates the differences between scRNA-seq and ST data in terms of the number of cells per cell type, and second it aligns the scRNA-seq dataset to the ST dataset via randomly sampling of the predicted number of cells for each cell type. Introducing placeholder cells, CytoSPACE maps each cell to spatial pixels by minimizing a correlation-based cost function. The limitation of this tool is that it depends on the fractional composition of each cell type per spot inferred by other tools such as robust cell type decomposition (RCTD) or Cell2location. Different from other tools, CytoSPACE can guarantee a globally optimal solution by convex linear optimization. CytoSPACE guarantees an optimal solution with convex optimization and is more noise-tolerant and computationally efficient, although it depends on the prior knowledge of cell type compositions and might be less flexible with biological assumptions.

In general, the choice of methods should be driven by the specific needs of the research. For instance, if the primary concern is biological realism and the data set is not excessively large, novoSpaRc or CellTrek could be preferable. If accuracy and the use of prior knowledge are paramount, Tangram may be the best choice despite its computational demands. For large datasets where an optimal and computationally efficient solution is required, CytoSPACE could be the most suitable option. It is also common for researchers to try multiple methods and compare results to choose the best approach for their particular dataset and research question. To sum up, the choice of methods largely depends on the specific research needs, dataset size, and availability of computational resources.

## Deconvolution-based algorithms for inferring cellular composition of ST data

Different from mapping-based algorithms, which maintain single-cell resolution but do not resolve the compositional disparity between the scRNA-seq and ST data, deconvolution-based algorithms seek to first preserve the compositional consistence, *i.e.*, reconstructing ST observations by assigning different weights to cell types derived from scRNA-seq data. These tools include RCTD [36], SPOTlight [37], Stereoscope [38], SpatialDWLS [an extension of dampened weighted least squares (DWLS)] [39], deconvoluting spatial transcriptomics data through graph-based convolutional networks (DSTG) [40], spatial transcriptomics deconvolution by topic modeling (STRIDE) [41], cell type inference by domain adaptation of single-cell and spatial transcriptomic data (CellDART) [42], Cell2location [43], DestVI [44], conditional autoregressive-based deconvolution (CARD) [45], Celloscope [46], and Redeconve [47] (Figure 2; Table S1).

### RCTD

RCTD applies a mixture model to infer the cellular composition at each spatial spot or pixel in spatially resolved transcriptomic data such as Slide-seq or 10X Genomics Visium datasets [36]. RCTD first calculates the mean expression of marker genes for each cell type derived from a scRNA-seq dataset. And then it builds a mixture model with the assumption of Poisson distribution to decompose each spatial pixel into combinations of different cell types by utilizing a maximum likelihood method. RCTD considers the gene-specific variance of platform effects and performs platform effect normalization to classify the cells across different platforms. RCTD assumes that platform effects are the same among different cell types, which may be improper in certain settings. Because of the computational complexity of mixture models, the number of cell types that RCTD can resolve may be upper-limited.

### SPOTlight

SPOTlight employs a seeded non-negative matrix factorization (NMF) regression to infer cell type-specific gene signatures in scRNA-seq datasets and relies on the non-negative least squares (NNLS) to obtain the cellular composition of spots [37]. The NMF algorithm from SPOTlight improves the sensitivity and robustness by applying the prior information to initialize the basis and coefficient matrices with cell type marker genes. In addition, SPOTlight uses a unit-variance normalization step to tolerate the technical disparity when matched or unmatched ST and scRNA-seq raw count matrices are provided as input.

### Stereoscope

Stereoscope is a probabilistic model-based method to conduct spatial spot deconvolution by using annotated scRNA-seq reference and ST data [38]. Unlike the other tools, Stereoscope uses complete expression profiles instead of a select set of marker genes as features. Stereoscope relies on the hypothesis that gene expression count matrix from both spatial and single-cell data follows a negative binomial distribution. When the cell types do not overlap perfectly in spatial and single-cell data, Stereoscope introduces “dummy” cell types to tolerate the disparity between ST and scRNA-seq data. Stereoscope can also handle extremely large datasets (> 1 million cells) by employing graphics processing unit (GPU) for fast inference.

### SpatialDWLS

SpatialDWLS first performs cell type enrichment analysis by using a parametric analysis of gene set enrichment (PAGE) method in each spot based on cell type signature genes derived from the scRNA-seq reference, and then uses a DWLS approach to estimate the cell type composition at each spatial location [39]. Compared to other deconvolution algorithms, the main difference of spatialDWLS lies in an additional filtering step to remove irrelevant cell types.

### DSTG

DSTG is a similarity-based semi-supervised graph convolutional network (GCN) model to infer cell type proportions in each spatial spot [40]. By integrating scRNA-seq data, DSTG first generates virtual mixtures of cells called “pseudo-ST spot” by randomly pooling two to eight cells selected from scRNA-seq data. The pseudo-ST spots constructed by combining scRNA-seq data of multiple cells form the basis for model training. Then, to measure the similarity between pseudo-ST spots and real ST data, DSTG employs a link graph by finding MNNs in the shared space identified by canonical correlation analysis (CCA). Next, a GCN is used on the link graph to propagate the real and pseudo-ST data in a latent space that is turned into a probability distribution of the cellular compositions for each spot. The cell type proportions can finally be displayed as a pie chart at each spatial spot. In summary, DSTG represents a class of algorithms that apply a synthesis strategy to simulate ST data based on scRNA-seq data and to infer the precise cellular composition of ST data based on a training and predicting framework.

### STRIDE

STRIDE is a topic-model-based deconvolution method for ST data analysis [41]. It decomposes cell types from spatial data via topic profiles learned from single-cell transcriptomics. STRIDE first employs topic modeling to obtain cell type-associated topics from well-annotated scRNA-seq datasets, and then applies the trained topic model to estimate the cell type compositions for each spatial location. The topic profiles generated by STRIDE are typically associated with functions defined by specific cell types, and thus could accurately reflect the spatial features of each cell type. Moreover, STRIDE has the capability to detect rare cell types from spatial spots by topics. The assumption underlying STRIDE, *i.e.*, the cell types and their compositions are similar between ST and scRNA-seq data, may also limit its performance when ST data are not well-matched with scRNA-seq data. Therefore, it is difficult for STRIDE to reveal the cellular combinations that are absent in scRNA-seq data but present in spatial data.

### CellDART

CellDART can predict the cellular compositions in ST data with different spatial resolutions by a modified adversarial discriminative domain adaptation (ADDA) algorithm [42]. ADDA is a successful deep learning algorithm in semantic alignment. The training of neural networks is first constructed based on TensorFlow, Keras, and scikit-learn algorithms. Similar to DSTG, CellDART constructs pseudo-spots based on randomly selected cells from scRNA-seq data, and the known fraction of cells in the pseudo-spots is used to train the neural networks. When the neural network model is well trained and learn the relationship between cell fraction and the gene expression profiles of pseudo-spots, the model can then be used to predict the cellular compositions of real spots from a different domain where ST data are present. The whole framework of CellDART is promising, especially when knowledge transfer across domains is needed.

### Cell2location

Cell2location utilizes a hierarchical Bayesian framework to predict cell type abundance in spatial data [43]. Cell2location first applies negative binomial regression to estimate cell type-specific signatures from scRNA-seq or snRNA-seq expression profiles or a set of user-defined cell types. Then, Cell2location used the estimated cell type signatures and ST matrix as input to decompose spatial compositions based on reference cell types. The key assumption of Cell2location is that the count matrix of ST data also follows a negative binomial distribution and can be decomposed into a linear combination of cell type signatures at individual spatial locations. Joint analysis of scRNA-seq and ST data is thus applied to learn the parameters defining the negative binomial distributions, and statistical strength across locations is integrated to improve the cell resolution of deconvolution analysis. Cell2location also considers batch variation across slides, messenger RNA (mRNA) detection sensitivity, and mRNA abundance of individual spots, which allow predicting the absolute abundance of different cell types.

### DestVI

DestVI applies a Bayesian model to simultaneously recover cell type proportions and cell type-specific snapshots of the transcriptional state based on scRNA-seq and ST data [44]. Unlike other methods, DestVI employs both discrete cell type-specific profiles and continuous sub-cell type latent variations based on a conditional deep generative model to recover the cell type frequency and the average transcriptional state of different cell types at each spatial spot. Because scRNA-seq data are generally produced from tissues different from those of ST data, expression differences exist and need to be reconstructed to best depict the specific spatial states of genes and cell types. The introduction of continuous cell states of different cell types by DestVI fulfills this type of analytical need and is better to explore the spatial context of gene expression.

### CARD

CARD performs the deconvolution analysis for ST by adding an assumption that spatially neighboring tissue locations have similar cellular compositions [45]. With this unique feature, CARD accommodates the spatial correlation structure across tissue locations by a conditional autoregressive (CAR) model in the deconvolution analysis. Generally, this assumption holds true for most of the spatial locations, and the inclusion of this assumption will greatly enhance the statistical power of deconvolution analysis. CARD tests the deconvolution analysis of ST data with the less matched scRNA-seq references and achieves performance similar to that of deconvolution analysis with well-matched reference, suggesting the robustness empowered by the newly introduced assumption. However, it should be noted that the correlation structure may break when orders among different spatial domains were analyzed, and thus the newly introduced hyperparameter because of this assumption needs to be finely tuned in practice at the special regions. CARD can also improve spatial resolution for ST data and conduct deconvolution analysis without scRNA-seq reference.

### Celloscope

Celloscope provides a probabilistic model for decomposing cell type mixtures in the spots from ST data based on established prior knowledge on marker genes [46]. Celloscope takes advantage of prior qualitative information on marker genes that are expected to be highly expressed in their respective cell types compared to other types. Additionally, Celloscope is characterized by the assumption that dummy types exist to account for cell types not covered by the marker genes. Large content of expert knowledge on marker genes for different cell types is available and can be readily obtained from the PubMed search engine or public databases such as CellMarker [48]. Celloscope can make use of these prior knowledge and is independent of scRNA-seq data. It should be noted that the encoding scheme of Celloscope may be critical to the analyzing results because of the qualitative nature of cell marker genes.

### Redeconve

Redeconve is a novel deconvolution tool used for analyzing ST data, which estimates the composition of cell states in each spot without needing to pre-cluster single cells [47]. It directly uses scRNA-seq or snRNA-seq data as a reference and introduces regularization to handle the challenge of similar gene expression profiles (collinearity) among cells. This method achieves higher consistency, resolution, and accuracy in estimating cellular composition compared to previous methods. Redeconve also offers fast computational speeds and can identify a high degree of cellular heterogeneity within tissue spots, aligning well with actual cell counts obtained through nucleus counting. Overall, Redeconve represents an advancement in the precise and efficient analysis of cellular compositions within spatially resolved tissue samples.

When selecting a method for deconvolution of ST data, researchers must consider various factors including the availability and match of scRNA-seq reference data, computational resources, spatial resolution requirements, the type of ST technology used, cell type diversity, batch effects, and prior biological knowledge. Methods like RCTD, SPOTlight, and Stereoscope require scRNA-seq data and differ in their approaches to handle large datasets, cell type heterogeneity, and spatial patterns. STRIDE is notable for detecting rare cell types, while Cell2location and DestVI are adept at accounting for technical variations and capturing cell state variations, respectively. CARD leverages spatial correlations, whereas Celloscope operates independently of scRNA-seq data by using prior knowledge on marker genes. The choice of methods ultimately hinges on the specific biological questions, data characteristics, and practical considerations such as ease of use and community support. Researchers often compare results across multiple methods to gain a robust understanding of their ST data.

## Unsupervised deconvolution algorithms

Previous algorithms require scRNA-seq data or at least cell marker genes as reference to analyze ST data, and thus could be categorized into supervised algorithms. Recently, unsupervised algorithms for ST data analysis emerged, *e.g.*, STdeconvolve [49] and BayesSpace [50] (Figure 2; Table S1). In principle, unsupervised algorithms can identify the hidden structure underlying ST data in an unbiased manner but cannot directly establish the relationship between ST data and cellular identity. In the authors’ perspective, defining cellular identity unsupervisedly may become feasible in the future when ST data accumulate sufficiently, and therefore these unsupervised algorithms represent an important direction of ST data analysis.

### STdeconvolve

STdeconvolve is an unsupervised machine learning approach that can deconvolve the spatial spots with multi-cellular resolution into putative ST profiles and their corresponding proportions within individual spots [49]. Technically, STdeconvolve employs latent Dirichlet allocation (LDA) to infer the putative transcriptional profiles and their proportions in each ST spot and interprets the inferred transcriptional profiles as cell types. Evaluations suggest that STdeconvolve can successfully reconstruct the transcriptional profiles for multiple major cell types, proving the validity of unsupervised deconvolution analysis. However, unsupervised deconvolution analysis may fail when limited ST data are available. With the coming flood of ST data in the future, unsupervised deconvolution analysis represented by STdeconvolve may further mature.

### BayesSpace

Similar to STdeconvolve, BayesSpace is also an unsupervised deconvolution algorithm, which employs a Bayesian model to increase the spatial resolution to the sub-spot level [50]. Different from STdeconvolve, which employs the global recurrent patterns of putative transcriptional profiles, BayesSpace enhances resolution based on the local information of ST spots. Evaluations suggest that BayesSpace can approach single-cell resolution on the 10X Genomics Visium platform, without the requirement of external single-cell data. With external information provided such as hematoxylin-eosin (H&E) staining, BayesSpace may be further improved to enhance spatial resolution of ST data.

In conclusion, unsupervised deconvolution algorithms like STdeconvolve and BayesSpace are pivotal for interpreting complex ST data. STdeconvolve employs LDA to deconvolve ST spots into putative transcriptional profiles, representing different cell types without needing external single-cell data. It excels in reconstructing profiles for various cell types, but its performance might decline with insufficient ST data. Conversely, BayesSpace uses a Bayesian model to enhance spatial resolution at a sub-spot level by exploiting local spatial information, aiming for single-cell resolution on platforms like 10X Genomics Visium, and can integrate histological stains for further improvement. While BayesSpace is powerful for increasing spatial resolution, its success is dependent on the quality of local information and additional data like histological alignment. The choice between these algorithms hinges on factors such as data availability, desired resolution, external data integration, computational requirements, and the specific research goals. Both methods are valuable in the field of ST, and their utility is expected to grow alongside the surge in available ST data.

## Outlook and challenges

Currently, spatial technologies can generally be categorized into two types, discriminated by multi-cellular and subcellular resolution (Figure 1). Subcellular resolution in ST refers to the ability to localize and quantify RNA molecules at the level of cellular organelles or specific regions within a cell. This high level of detail is crucial for understanding the spatial heterogeneity of gene expression, how it contributes to cell function and organization, and how it may change in various states of health and disease. To achieve subcellular resolution in ST, several techniques and advancements have been developed, including fluorescence *in situ* hybridization (FISH), *in situ* sequencing (ISS), MERFISH, sequential FISH (seqFISH), and STARmap. By integrating these advanced imaging and molecular biology techniques, subcellular-resolution ST can provide insights into the functional organization of the cell, reveal the spatial dynamics of gene expression during different cellular processes, and uncover the cellular architecture of complex tissues. This fine-grained spatial information is invaluable for unraveling the complexities of tissue development, cellular differentiation, and disease progression.

**Figure 3:**
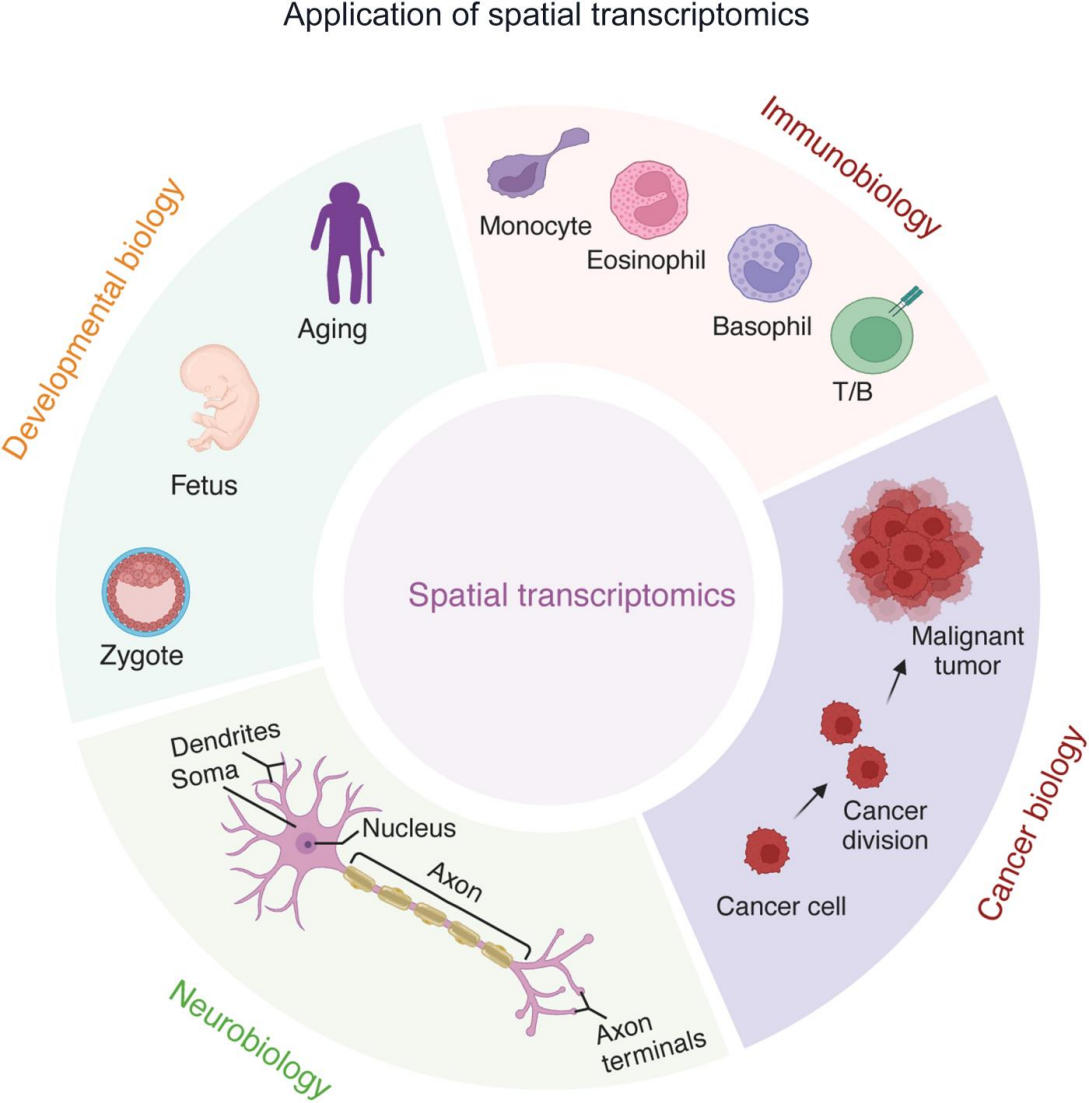
Potential application of spatial transcriptomics The potential application of spatial transcriptomics includes developmental biology, immunobiology, cancer biology, and neurobiology. This figure is created with BioRender.com.

Because spatial technologies with multi-cellular resolution can generally obtain the transcriptional profiles of the whole transcriptome, they are important to studies with the aim to identifying novel genes with specific spatial features or related to specific phenotypes, potentially in developmental biology, immunobiology, cancer biology, and neurobiology (**Figure 3**). Moreover, multi-cellular spatial technologies generally require less cost than subcellular technologies. Therefore, deconvolution analysis of this type of ST data is in need and of broad interest. Most of the currently developed methods require additional scRNA-seq data or cell markers as reference to recover the cellular identity of ST data via mapping-based or deconvolution-based strategies, while a few algorithms start to deconvolve ST data in an unsupervised manner. Ideally, the reference data should be generated from the same tissue as the ST data are produced. In practice, this requirement may be difficult to meet, and public scRNA-seq data from the same tissue type may also be acceptable as a compromised solution. Overall, the current algorithms have provided effective decomposition of the cellular content of ST data, enabling further data mining and resolving biomedical questions. Mapping-based algorithms have the advantage to provide single-cell resolution, but the proportions and absolute cell numbers may be questionable because of the disparity between ST and scRNA-seq data. Deconvolution-based algorithms have the advantage to faithfully reconstruct the ST data with different cellular compositions, but the resolution is currently limited.

Deconvolution at single-cell resolution is of pressing need to fully mine the power of ST data. The basic idea behind single-cell deconvolution is to decompose the mixed expression signals obtained from each spot into contributions from different cell types. This involves using reference scRNA-seq datasets to generate gene expression profiles for each cell type. By comparing these profiles with the ST data, the proportion and location of different cell types within the tissue can be inferred. The challenges of single-cell deconvolution for ST data can be summarized as follows. (1) Resolution mismatch. ST often captures gene expression from multiple cells in a single spot due to lower resolution compared to single-cell methods, which can lead to mixed signals that are difficult to attribute to individual cells. (2) Reference data limitation. Deconvolution methods typically require well-annotated reference scRNA-seq datasets to provide the cell type-specific expression profiles necessary for decomposing mixed signals, and the lack of such datasets for certain tissues or conditions can be a major limitation. (3) Validation. Validating the results of deconvolution can be difficult, especially in the absence of ground truth or complementary datasets such as *in situ* hybridization or immunohistochemistry. Overall, while single-cell deconvolution is a powerful approach for understanding the cellular composition and spatial organization of tissues, it faces significant challenges that must be addressed to ensure accurate and reliable results. Advances in computational methods, as well as improvements in ST technologies, are helping to overcome these challenges. Yang Can’s lab (The Hong Kong University of Science and Technology, China) developed a unified approach for integrating spatial and single-cell transcriptomics data that leverages deep generative models [51]. It uses a deep generative model to capture complex patterns in the data, which not only enhances sequencing-based ST data to achieve single-cell resolution but also accurately infers transcriptome-wide expression levels for image-based ST data, potentially handling a wide variety of tissue types and conditions. Recently, we developed a deconvolution-based algorithm for multi-cellular ST data analysis, named as Redeconve, which introduces a novel assumption which hypothesizes that cells with similar phenotypes may have similar abundance at the same spatial locations [47]. Evaluations on multiple ST datasets proved the effectiveness of Redeconve, suggesting the feasibility of deconvolution at single-cell resolution. Different from multi-cellular ST data, the analysis of subcellular ST data requires cell segmentation, which requires merging different spots into one cell and is thus mathematically a reverse question of deconvolution analysis. The success of mapping and deconvolution algorithms for multi-cellular ST data may provide important insights into the development of cell segmentation algorithms.

The improved accuracy of deconvolution methods in ST can significantly enhance several downstream analyses, thereby providing deeper insights into tissue architecture, disease mechanisms, and cellular interactions. Here are some downstream analyses that can benefit. (1) Cell type-specific gene expression analysis: accurate deconvolution can provide more reliable estimates of cell type-specific gene expression patterns, enabling more precise identification and characterization of cell types within the spatial context. (2) Cell–cell interaction analysis: deconvolution can help in identifying and quantifying interactions between different cell types within a spatial tissue context, providing insights into cellular communication networks and signaling pathways. (3) Spatial gene expression analysis: improved deconvolution accuracy can enhance the spatial resolution of gene expression patterns, enabling more accurate reconstruction of tissue architecture and identification of spatially restricted gene expression patterns. (4) Biomarker identification: accurate deconvolution can aid in the identification and validation of cell type-specific biomarkers, which can have implications for disease diagnosis, prognosis, and therapeutic targeting. These are just a few examples, and there may be other downstream analysis techniques and applications that can benefit from improved deconvolution accuracy. To harness these benefits, ongoing research and development in deconvolution methods are necessary to ensure that the downstream analyses are based on robust and accurate cellular resolution of complex tissue environments.

In conclusion, the deconvolution of ST data provides an insightful glimpse into the cellular architecture of complex tissues. However, the prevalence of noise necessitates cautious interpretation of the results. To ensure that our conclusions are both statistically sound and biologically relevant, it is essential to engage in rigorous validation and continual refinement of our methodologies. Noise in ST data arises from both technical artifacts and biological variability, which can significantly undermine the precision of deconvolution analyses. Technical noise, such as batch effects and variations in sequencing depth, can lead to erroneous attribution of gene expression differences to cell populations instead of technical inconsistencies. Biological noise, including cell heterogeneity and rare cell types, can cause the deconvolution algorithms to overlook subtle but important cellular patterns or falsely identify cell populations. These challenges underscore the risk of misleading outcomes, where the estimated proportions and identities of cell types in a tissue may not accurately reflect the true biological scenario. To minimize these risks, robust computational methods, careful experimental design, and thorough validation strategies are imperative for obtaining reliable deconvolution results that truly enhance our understanding of tissue architecture and function.

## CRediT author statement

**Xiuying Liu:** Writing – original draft, Writing – review & editing. **Xianwen Ren:** Conceptualization, Writing – original draft, Writing – review & editing. Both authors have read and approved the final manuscript.

## Supplementary Material

qzae057_Supplementary_Data
