# Supplementary material for: Computational Strategies and Algorithms for Inferring Cellular Composition of Spatial Transcriptomics Data
Source: Genomics Proteomics Bioinformatics. 2024 Aug 7;22(3):qzae057. doi: 10.1093/gpbjnl/qzae057 (PMC11398939; doi:10.1093/gpbjnl/qzae057)
Supplement: qzae057_Supplementary_Data [file qzae057_supplementary_data.zip › supplementary material captions.docx]

**Supplementary material**

**Table S1 An overview of ST decomposed methods**
